# Insight into the Antioxidant Activity of Ascorbic Acid-Containing Gelatin Nanoparticles in Simulated Chronic Wound Conditions

**DOI:** 10.3390/antiox13030299

**Published:** 2024-02-28

**Authors:** María del Carmen Morán, Cristina Porredon, Coloma Gibert

**Affiliations:** 1 Departament de Bioquímica i Fisiologia-Secció de Fisiologia, Facultat de Farmàcia i Ciències de l’Alimentació, Universitat de Barcelona, Avda. Joan XXIII, 27-31, 08028 Barcelona, Spain; cporredon@bellvitgehospital.cat (C.P.); cgibertsalva@gmail.com (C.G.); 2 Institut de Nanociència i Nanotecnologia-IN2UB, Universitat de Barcelona, Avda. Diagonal, 645, 08028 Barcelona, Spain

**Keywords:** antioxidant, ascorbic acid, chronic wound, gelatin, hydrogen peroxide, pH

## Abstract

Chronic wounds differ from acute wounds by remaining in the inflammatory phase for a long time. This chronic inflammation confers a high concentration of inflammatory cytokines, proteases, and ROS. Likewise, the pH environment of chronic wounds has been recorded within the range of 7.2–8.9 due to the alkaline by-products of bacterial proliferation. In this work, differences in pH between healthy skin and chronic cutaneous wounds have been used for the design and development of pH-responsive gelatin-based nanoparticles (NPs). Ascorbic acid (AA), as an antioxidant compound that can neutralize reactive oxygen species (ROS), has been the therapeutic model compound included in these NPs. The goal of the present work has been the preparation and characterization (physicochemical and biological properties) of NPs for the effective release of AA under simulated chronic wound conditions. In vitro experiments demonstrated total AA release at pH corresponding to the chronic wounds. The biocompatible character of these gelatin-based NPs based on their hemolytic and cytotoxicity responses has been highlighted under in vitro conditions. The reversible and protective antioxidant properties of the AA-including NPs in erythrocytes and skin cell lines, respectively, have been confirmed to be modulated by the gelatin A gel strength.

## 1. Introduction

Knowledge about the biology of wound healing has evolved significantly in the last 20 years. Nowadays it is possible to predict the likely sequence of events that will take place throughout healing and predict the approximate time in which a wound will close completely [1]. However, despite the growing availability of information and the development of numerous interactive wound care products, healthcare professionals will find wounds in which healing will be prolonged or not achieved. A wound is considered chronic when it shows no or minor signs of healing after three months [2].

Different factors contribute to chronicity. Age, malnutrition, drugs such as immunosuppressants or glucocorticoids, alcohol and tobacco consumption are important parameters at the systemic level. One of the consequences of the increase in life expectancy is the increase in chronic diseases, and among them, chronic injuries must be considered. Factors such as vascular diseases, neuropathies or pressure, infections, necrotic tissue or excessive pressure on the wound, an unfavorable local environment, repetitive trauma, and radiation stand out [3]. It is expected that 1–2% of the total population will experience a chronic wound during their lifetime, this incidence being threefold for older people (>75 years). From an economic point of view, by 2032, the global wound care market is expected to reach USD 37.3 billion from USD 21.2 billion in 2022 [4].

Chronic wounds differ from acute wounds by remaining in one or more of the wound-healing phases. Among them, the inflammatory phase persists for a long time. Due to this chronic inflammation, these wounds are characterized by a high concentration of inflammatory cytokines, proteases, and reactive oxygen species (ROS), triggering a degraded matrix. High levels of bacterial load, low mitosis activity, and cell senescence are other features [5]. Free radicals play a critical role in healing, acting in different stages, such as in coagulation and platelet aggregation, as well as mediators of the inflammatory response or in reepithelialization [6]. When there is an imbalance between the production of free radicals and the body’s antioxidant capacity, oxidative stress occurs. Excess of ROS leads to fibroblast senescence, making it challenging to synthesize the extracellular matrix necessary for cell migration and healing. 

Ascorbic acid (AA) is an excellent antioxidant with the ability to neutralize ROS. AA is an essential compound for the synthesis of collagen and other organic components of the intracellular matrix of tissues such as bones, skin, and other connective tissues [7]. It is also involved with normal responses to physiological stressors such as in accidents and surgical trauma and the need for AA increases during times of injury [8]. AA also improves immune function, particularly during infection [9,10]. It has long been used in pharmaceutical and cosmetic formulations concerning its beneficial effects on the skin. However, its low stability is a severe limitation: it is an easily oxidizable compound, especially under aerobic conditions and exposure to light, degraded in a first step reversibly to dehydroascorbic acid and then to oxalic acid in an irreversible manner [11].

Drug research focuses on improving controlled release and specific targeting. Nanoparticles (NPs) are encapsulation systems used to improve therapeutic agent efficacy, such as antiviral agents, anticancer agents, or diagnostic [12,13], with minimal toxicity. For this reason, the design and development of methods that can effectively generate these NPs are required. In recent years, there has been increasing effort to develop stimuli-responsive nanomaterials that will be established into effective drug delivery systems (DDS) [14]. The success of these DDS is their capability to respond to endogenous or exogenous stimuli to promote local responses and target the delivery of the drug. Often, their efficiency is correlated with their capability for nanomaterial-facilitated accumulation and/or cellular internalization [15]. 

Wound pH is an indicative parameter for assessing the healing process of chronic wounds. Unlike acute wounds, which have a slightly acidic pH (5.5–6.5), chronic ones often present values ranging from 7.2 to 8.9 due to the alkaline by-products of bacterial proliferation. In addition, the irregular vascular structure of chronic wounds causes a heterogeneous distribution of infection, so there are drastic pH variations in the affected area [16]. NPs that are not easily degraded and can circulate could be obtained using hydrophilic and flexible polymers [14], as is the case of gelatin. Gelatin NPs can be prepared by several techniques, including desolvation, coacervation-phase separation, emulsification-solvent evaporation, reverse-phase microemulsion, and nanoprecipitation. The preparation methods of gelatin NPs have been elaborated and revised [17,18]. Using these methods, the obtained materials need to be cross-linked to improve their poor mechanical properties and control their rate of biodegradation. Biological methods (genipin, transglutaminase and plant-derived proanthocyanidins) have demonstrated improved biocompatibility in comparison with chemical treatments using glutaraldehyde, carbodiimide or N-hydroxysuccinimide [19].

Recent studies in our research group have allowed the generation of new gelatin-based NPs based on the interaction between oppositely charged compounds to encapsulate and control the release of molecules of therapeutic interest such as DNA, RNA, and antitumor drugs [20,21,22,23]. The gelation properties of gelatin and its degree of ionization as a function of pH make this compound an exciting alternative for preparing vehicles for the encapsulation and selective release of therapeutical molecules.

**Scheme 1:**
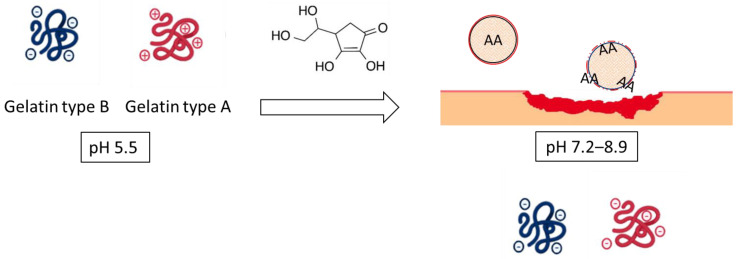
Synthesis of gelatin-based NPs based on the interaction of oppositely charged compounds between gelatin type B and gelatin type A.

In the present work, differences in pH between healthy skin and chronic wounds have been projected to design and develop stimuli-responsive NPs. Specifically, the formation of NPs is based on the interaction of oppositely charged compounds between gelatin type B (GB) (pI 4.8–5.2) and gelatin type A (GA) (pI 7–9, as a function of gel strength) [24] at the pH of healthy skin. The subsequent increase of pH up to 7, representative of non-healing wounds, would promote the charge inversion of GA. The repulsion between the negative charges of both proteins would facilitate the destabilization of the NPs system and the consequent release of the encapsulated AA (Scheme 1).

Using this hypothesis, gelatin-based NPs may provide an opportunity to increase the therapeutic effect of AA under chronic wound conditions using multiple approaches: (1) selectively protecting and releasing the cargo using the charge reversal approach on the surroundings of the chronic wound and (2) promoting the adhesion and proliferation of senescence fibroblasts and keratinocytes by stimulation of ECM production mediated by gelatin-based carrier.

This work aims to prepare and characterize (physicochemical and biological properties) AA-containing NPs for effective release under chronic wound conditions. The physicochemical characterization (particle size, polydispersity index, degree of AA entrapment, and AA release) has been evaluated as a function of both GB and AA concentration and GA gel strength (either low or high blooms values) at the representative pHs of healthy skin and chronic wound conditions. In vitro experiments have been performed to determine the biocompatible characterization of these gelatin-based NPs regarding their hemolytic and cytotoxicity properties at those representative pHs. Due to the impact of ROS production on the chronicity of the wounds, the antioxidant efficiency of the AA-containing NPs has been evaluated in both erythrocytes and representative skin cell lines (3T3 and HaCaT) in comparison with AA in solution, as a function of pH.

## 2. Materials and Methods

### 2.1. Materials

Gelatin from bovine skin (gelatin type B) with gel strength 75 bloom value (GB), gelatin from porcine skin (gelatin type A) with gel strength values ranging between 100 (GA100), 175 (GA175), and 300 bloom values (GA300), and ascorbic acid (AA) were purchased from Sigma-Aldrich ((St. Louis, MO, USA). and used as received. 2,2′-Azobis(2-amidinopropane) dihydrochloride (AAPH) and hydrogen peroxide (H_2_O_2_) were purchased from Sigma Aldrich ((St. Louis, MO, USA). and used as received. Phenol-indo-2,6-dichlorophenol (DPIPC) was purchased from Across Organics (now ThermoFisher Scientific, Geel, Antwerp, Belgium) and used as received. 

Dulbecco’s Modified Eagle’s Medium (DMEM), fetal bovine serum (FBS), L-glutamine solution (200 mM), penicillin-streptomycin solution (10,000 U/mL penicillin and 10 mg/mL streptomycin), phosphate-buffered saline (PBS), and trypsin-EDTA solution (170,000 U/L trypsin and 0.2 g/L EDTA), were purchased from Lonza (Verviers, Belgium). 2,5-Diphenyl-3,-(4,5-dimethyl-2-thiazolyl) tetrazolium bromide (MTT) and dimethylsulfoxide [(DMSO) were obtained from Sigma–Aldrich (St. Louis, MO, USA). The 75 cm^2^ flasks and 96-well cell culture plates were obtained from TPP (Trasadingen, Switzerland). All other reagents were of analytical grade.

### 2.2. Methods

#### 2.2.1. Preparation of Gelatin and AA Solutions

Solutions of GB, AA, and GA were prepared by dissolution in PBS pH 5.5 buffer. GB was prepared at concentrations ranging from 10 to 40 mg/mL. A stock solution of AA at 10 mg/mL was prepared, and solutions with 2.5 and 5 mg/mL concentrations were obtained by subsequent dilution. The GA concentration was fixed at 0.1 mg/mL.

#### 2.2.2. Preparation of GB(AA)binary Mixtures

AA was incorporated into the gelatin network when GB and AA solutions, previously prepared in PBS pH 5.5, were mixed at a ratio equal to 50% (*v*/*v*). Consequently, the final concentration of GB and AA was fixed to 5–20 mg/mL and 1.25–5 mg/mL, respectively. Gel formation was promoted by placing the obtained mixtures in the refrigerator overnight.

#### 2.2.3. Preparation of GB (AA)-GA NPs

Binary mixtures containing GB and AA (200 µL) were added dropwise into the GA solution (2 mL) under gentle stirring. Under optimal conditions, droplets from the mixed GB (AA) systems broke under magnetically stirring and instantaneously evolved into discrete NPs in contact with the GA solution (0.1 mg/mL) by the interaction between oppositely charged compounds (GB and GA). For comparative purposes, NPs in the absence of AA were also prepared. NPs remained in the GA solution for 1 h to favor the contact between oppositely charged compounds.

#### 2.2.4. Physicochemical Characterization of the GB(AA)-GA NPs

1. Size and polydispersity

Dynamic light scattering [DLS] by the Zetasizer Nano ZS90 was used to determine the size of the obtained NPs. The size distribution was assessed from the polydispersity index (pdI), ranging from 0.0 for an entirely monodisperse sample to 1.0 for a polydisperse sample. The size distribution by the intensity of scattered light was used for the data interpretation. Three different measures were performed for each condition. Every single measure consists of 10 sub-measures of 10 s at 25 °C.

2. Loading Efficiency

The degree of AA entrapment was determined through the loading efficiency (LE) values, following the equation:

LE (%) = [Encapsulated AA]/[Total AA] × 100(1)

where

[Encapsulated AA] = [Total AA] − [free AA] (2)

The amount of free AA was determined using the DCPIP method. This method is based on the redox reaction between phenol indophenol (DCPIP) and ascorbic acid (AA). When evaluating free AA in solution, it is transformed into dehydroascorbic acid (DHA) and DCPIP changes from oxidized to reduced form, showing a change in color from blue to colorless. 

A special effort has been made in this work to establish a protocol that allows applying the DCPIP method at the milliliter scale. Thus, 670 µL of DCPIP was mixed with 330 µL of PBS pH 5.5 solution to establish the maximum absorption wavelength (Shimadzu UV-160A). The DCPIP-AA calibration curve was prepared using eight AA concentrations ranging between 0 to 5 mg/mL in PBS pH 5.5. The absorbance of the resulting solutions was determined at a wavelength of 590 nm at room temperature. The free AA concentration in the NPs dispersions was determined by mixing of 670 µL of DCPIP with 330 µL of the NPs dispersions. The assay included bare NPs (prepared in the absence of AA) to verify any interference in the assay due to the presence of the NPs.

The effect of increased pH from 5.5 to 7.4 and 9.0 mimicking chronic wound conditions on LE values was also determined. After successive alkalization by adding the required volumes of 0.5 M NaOH solution and incubation for 1 h, aliquots of supernatant were subsequently removed to quantify the free AA in the solution. The effect of pH increase was evaluated through the release efficiency (RE) following the equations:

RE (pH 7.4) (%) = (LE (pH 5.5) − LE (pH 7.4))/LE (pH 5.5) × 100(3)

RE (pH 9.0) (%) = (LE (pH 5.5) − LE (pH 9.0))/LE (pH 5.5) × 100(4)

DCPIP-AA calibration curves were performed at pH 7.4 and 9.0. Curves were strongly dependent on pH and DCPIP concentration (Supplementary Material Figure S1).

#### 2.2.5. In Vitro Assay with Erythrocytes

1. Obtention and extraction of the erythrocytes

Human blood samples were obtained from the Banc de Sang i Teixits de Barcelona (Blood and Tissue Bank) from the Catalan Department of Health. Blood was deposited in tubes with anticoagulant EDTA-K3. Blood samples were centrifugated at 3000 rpm at 4 °C for 10 min (Megafuge 2.0 R. Heraeus Instruments, Hanau, Germany) to induce sedimentation. Plasma was extracted with a Pasteur pipette. The residual pellet was washed with PBS pH 7.4. This procedure was repeated three times to remove residual leukocytes and platelets and to concentrate the erythrocytes. Following the last wash, the erythrocytes suspension was diluted (1:1) in PBS pH 7.4 to obtain suitable erythrocytes suspension (cell density of 8 × 10^9^ cell/mL).

2. Hemolysis assay

A hemolysis assay determines the capability of the NP systems to induce lysis of the erythrocyte membrane. Different NPs volumes (100, 200, and 300 µL) were placed in polystyrene tubes and an aliquot of 25 µL of erythrocyte suspensions was added to each tube. The final volume was 1 mL. The tubes were incubated at room temperature by rotatory conditions. After that, the tubes were centrifugated at 10,000 rpm for 5 min. The supernatant absorbance at 540 nm (Shimadzu UV-160A) was compared with those of the control samples hemolyzed with distilled water (positive control). Negative control was obtained by incubating an aliquot of 25 µL of the erythrocyte suspension with PBS pH 7.4.

3. Agglutination assay

An agglutination assay determines the putative aggregation of erythrocytes when they are incubated in the presence of the NPs system. After the hemolysis assay, 100 µL of the erythrocyte suspension was fixed with 100 µL of formaldehyde 4% and kept at 5 °C until it was studied. Then, the preparation was diluted by the addition of 200 µL of PBS pH 7.4. A small volume of this preparation (10 µL) was put on a slide and fixed by a coverslip. After that, the sample was studied with an optical microscope with phase contrast (Olympus BX4) using the 40× objective. The images were digitalized using a camera (Olympus digital camera XC50) and analyzed by an image processor (Cell B analysis).

4. Reversible antioxidant activity of NPs against peroxidation induced by AAPH

Hemolysis is induced with peroxyl radicals released by 2,2′-azobis-(amidinopropane) dihydrochloride (AAPH). The addition of AAPH to the erythrocyte suspension induces the oxidation of lipid and protein cell membranes inducing hemolysis. A typical assay involves incubating 250 µL of the erythrocyte suspension in the presence of AAPH (250 µL) at a final concentration of 100 mM for 150 min at 37 °C to achieve 80% hemolysis. The ability of NPs to reverse the effect of AAPH induced in erythrocytes was determined by incubation with 500 µL of the NPs dispersion. Similarly, the antihemolytic capacity of AA in solution was determined at the same concentrations as those included in the NPs. In all cases, the samples were incubated for 150 min at 37 °C and then centrifuged at 10,000 rpm for 5 min. The degree of hemolysis was determined by comparing the absorbances of the supernatants at 540 nm (Shimadzu UV-160A, Shimadzu, Duisburg, Germany)) with the positive and negative control, obtained by incubating 25 µL of the erythrocyte suspension with distilled water and PBS buffer 7.4, respectively.

#### 2.2.6. In Vitro Assay with Cell Cultures

1. Cell cultures

The murine Swiss albino fibroblast (3T3) and the immortal human keratinocyte (HaCaT) were obtained from Celltec UB (Barcelona, Spain). Cells were grown in DMEM medium (4.5 g/L glucose) supplemented with 10% (*v*/*v*) FBS, 2 mM L-glutamine, 100 U/ mL penicillin, and 100 µg/mL streptomycin at 37 °C, 5% CO_2_. Cells were routinely cultured in 75 cm^2^ culture flasks and were trypsinized using trypsin-EDTA when the cells reached approximately 80% confluence.

2. Cell viability assays

3T3 and HaCaT cells (1 × 10^5^ cells/ mL) were grown at the defined densities into the central 60 wells of a 96-well plate. Cells were incubated for 24 h under 5% CO_2_ at 37 °C. Then, the spent medium was removed, and cells were incubated for 24 h with the corresponding NPs systems, previously diluted 50% (*v*/*v*) in DMEM medium supplemented with 5% FBS (100 µL). The influence of pH values close to those representatives of chronic wound conditions was simulated by incubation of the NPs dispersion previously treated with suitable volumes of NaOH 0.5 M to achieve pH 7.4 and 9.0. 

Only living cells can reduce the yellow tetrazolium salt, 2,5-diphenyl-3,-(4,5-dimethyl-2-thiazolyl) tetrazolium bromide (MTT) to insoluble purple formazan crystals. After 24 h of incubation of the cells with the corresponding NPs, the medium was removed, and 100 µL of MTT (Sigma–Aldrich) in PBS (5 mg/mL) was diluted 1:10 in culture medium without phenol red and the absence of FBS (Lonza, Verviers, Belgium)) was added to the cells. After 3 h of incubation, the medium was removed. Thereafter, 100 µL of DMSO (Sigma–Aldrich St. Louis, MO, USA) was added to each well to dissolve the purple formazan crystals. Plates were placed in a microtiter-plate shaker for 5 min at room temperature to facilitate the total dissolution. The Bio-Rad 550 microplate reader was used to measure at 550 nm the absorbance of the resulting solutions. The effect of each treatment was calculated as the percentage of dye uptake by viable cells against the control cells (cells without any treatment). The induction of proliferation of the NP systems was evaluated through the stimulation index [25]:

SI = Cell viability induced by NPs/Cell viability in control cells (5)

3. Fluorescence microscopy

According to the standard procedure, changes in the growth and morphology of cells induced by NPs were evaluated using the double staining acridine orange/ethidium bromide (AO/EB). Cells were seeded at the above-mentioned densities into 24-well tissue plates on Corning’s circular glass coverslips at 37 °C and 5% CO_2_ atmosphere. After incubation for 24 h, cells were treated with 500 µL of each system diluted 1:1 in fresh medium supplemented with 5% FBS was added to each well. After 24 h incubation, the medium was removed, and the fluorescent dyes AO (0.5 μg/mL) and BE (10 μg/mL) were added. Next, the plates were incubated for a further 2 h at 37 °C and 5% CO_2_ atmosphere, after which the medium was removed, and the cells were washed four times with sterile PBS. After the final wash, the cells were fixed with 4% (*v*/*v*) paraformaldehyde in sterile PBS for 15 min at room temperature and were washed twice with sterile PBS. The coverslips were then mounted on clean glass slides with Prolong^®^Gold antifade reagent (Invitrogen, Waltham, MA, USA). Fluorescence images were acquired with an Olympus BX41 microscope equipped with a UV-mercury lamp (100 W Ushio Olympus, Tokyo, Japan) and a filter set type a U-N51004v2-FlTC/TRITC-type filter set (FITC: BP480-495, DM500-545, BA515-535, and TRITC: BP550-570, DM575-, BA590-621). Images were digitized on a computer through a video camera (Olympus digital camera XC50, Tokyo, Japan) and were analyzed with an image processor (Cell B analysis).

4. Protective antioxidant activity of NPs against oxidative stress induced by hydrogen peroxide

The protective capacity of the NPs to induce oxidative stress was evaluated when cells were subjected to two different hydrogen peroxide concentrations. Concentrations equal to 1 and 4 mM H_2_O_2_ were chosen after assaying the induced cytotoxicity of this compound in both cell lines. After 24 h of incubation of the cells with the corresponding NPs, the medium was removed, and the required volume of hydrogen peroxide was added. After 2.5 h of incubation, the MTT assay was performed under the above-described conditions. The antioxidant capacity of the NP systems was assessed through the percentage of the protective capacity (PC) of the cytotoxicity induced by hydrogen peroxide [26]:

PC (%) = (Cell viability induced by NP-cell viability induced by H_2_O_2_)/(Cell viability induced by NPs) × 100 (6)

#### 2.2.7. Statistical Analyses

Experiments were performed three times on independent occasions unless otherwise stated. The results are expressed as means standard deviation. One-way analysis of variance (ANOVA) was used to evaluate statistical differences between data sets, followed by Scheffé post hoc tests for multiple comparisons. IBM SPSS Statistics software version 29.0 was used to execute statistical analyses. Differences were considered statistically significant at *p* < 0.001. Significant differences were highlighted in the figures with an asterisk or other superscript symbols.

## 3. Results

### 3.1. Physicochemical Characterization of GB (AA)-GA NPs

The working hypothesis is based on the formation of NPs by the interaction of oppositely charged compounds at pH 5.5. GB remains negative at this pH due to its isoelectric point (pI 4.7), whereas GA (pI 7–9) may exhibit a positive charge. AA shows a negative charge at the assayed pH (pKa: 4.25).

**Figure 1:**
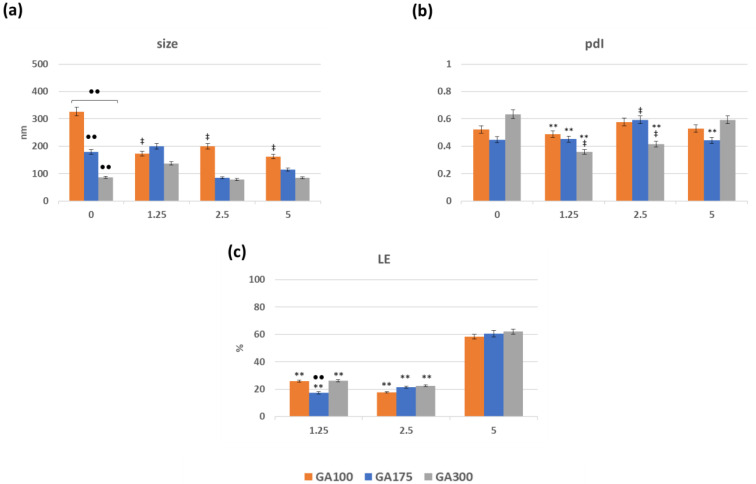
Effect of the imposed compositions on the size (**a**), pdI (**b**) and loading efficiency of AA (**c**) of GB(AA)-GA NPs prepared at the highest GB concentration (20 mg/mL). Results are expressed as the average of three independent experiments ± standard deviation. ** (*p* < 0.001) indicates significant differences between AA concentration for the same GA gel strength. ^●●^ (*p* < 0.001) indicates significant differences between GA gel strength values for the same AA concentration. ^‡^ (*p* < 0.001) indicates significant differences between NPs in absence and presence of AA.

The preparation of the GB(AA)-GA NPs consists of adding highly viscous GB (AA) binary mixtures to GA solutions with vigorous stirring. In this series of experiments, three variables were considered: (i) GB concentration (ranging from 2.5 to 20 mg/mL), (ii) AA concentration (ranging from 1.25 to 5 mg/mL), and (iii) GA gel strength (either 100, 175 and 300 gel bloom) at 0.1 mg/mL. For comparative purposes, NPs in the absence of AA (bare NPs) were also prepared. The resulting NPs were characterized by several physiochemical parameters such as size, polydispersity index, and loading efficiency (Supplementary Material Figure S2). Representative results of GB(AA)-GA NPs prepared at the highest GB concentration (20 mg/mL) are shown in Figure 1. Optimal size values ranged between 90 and 300 nm (Figure 1a). The size of bare GA100NPs demonstrated significant differences from those corresponding with GA175NPs and GA300NPs. AA-containing GA100NPs showed reduced size in comparison with bare NPs with significant differences. When the effect of AA concentration for the same GA gel strength was evaluated, significant differences were found between bare and AA-containing GA100NPs. However, no significant differences between NPs sizes at different AA concentrations were found. For GA175NPs and GA300NPs, neither difference between bare and AA-containing NPs or NPs at different concentrations were found.

Generally, pdI values range from 0.3 to 0.6, confirming the moderate polydispersity of the NPs (Figure 1b). In all cases, significant differences were obtained for NPs prepared at different gel strength values for the same AA conditions. The presence of AA in the NPs induces significant differences between different AA concentrations for the same GA gel strength. 

One of the crucial parameters of a DDS is its ability to encapsulate the drug of interest. The degree of encapsulation of AA in the NPs was determined through the loading efficiency (LE) values, considering the amount of non-encapsulated AA The lack of stability of free AA in solution was evident due to the sharp decrease in the signal at 266 nm, and the appearance of a signal below the UV–VIS absorption spectrum, probably dehydroascorbic acid [27]. For this reason, the reagent DCIPIP, which acts as a redox pair in the presence of AA was used to determine the free AA in solution [28,29]. Figure 1c summarizes the obtained results concerning the LE values of the GB(AA)-GA NPs. LE values varied between 17 and 62%, depending on the imposed compositions. LE values strongly depend on the initial AA concentration, promoting maximum LE values for the systems prepared with the highest AA concentration. Significant differences between AA concentrations for the same GA gel strength value were obtained. 

### 3.2. In Vitro Release from GB (AA)-GA NPs: Effect of the Alkalization of the Medium Mimicking Chronic Wounds Environment 

Since the electrostatic interaction between GB and GA is the main driving force for the preparation of these NPs, an increase in pH, such as what happens in chronic wounds, would influence the stability of NPs leading to the carrier destabilization and AA release.

**Figure 2:**
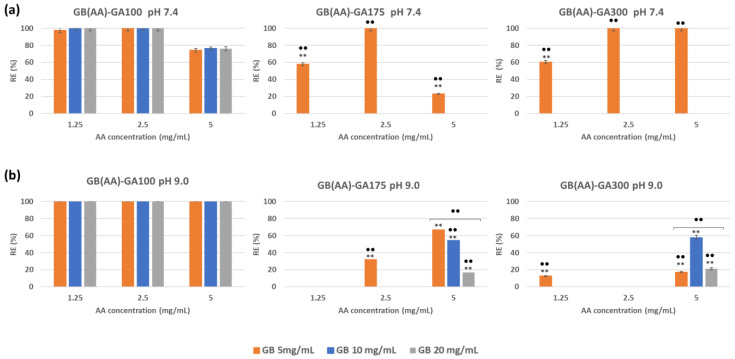
Effect of increased pH to 7.4 (**a**) and 9.0 (**b**) on the efficiency of AA release from GB(AA)-GA NPs as a function of GA gel strength and GB concentration. Results are expressed as the average of three independent experiments ± standard deviation. ** (*p* < 0.001) indicates significant differences between AA concentration for the same GB concentration. ^●●^ (*p* < 0.001) indicates significant differences between GB concentration for the same AA concentration.

To evaluate the AA release from GB (AA)-GA NPs, the release efficiency (RE) ratio was used, comparing the LE values at pH 7.4 and 9.0 with those obtained at pH 5.5. Figure 2a shows representative RE results at both pHs. A quick look at the RE responses demonstrated that pH but also GA gel strength (and their relationship with pI are controlling parameters of the in vitro release responses from these gelatin-based. At pH 7.4, the efficacy of AA release strongly differed with GA gel strength, with values close to 100% for GA100 NPs, due to the charge inversion of GA100 (pI close to 7) at those pH values. No significant differences between GB concentrations for the same AA concentration were found.

The increase of GA gel strength to 175 or 300 blooms (GA175 or GA300) promotes more limited release. At pH 7.4, when the pI of those gelatins seems to be not achieved, RE values may be related to leakage of poor incorporated AA from NPs at the lowest GB concentration. The efficacy of AA release could be 0% for NPs prepared with GB concentrations equal to 10 and 20 mg/mL. NPs prepared at the lowest concentration of GB (5 mg/mL) enable the AA release, with values ranging between 30 and 100%. These results could be explained by considering the more limited incorporation of AA into the corresponding 3D network of GB. Drugs became more efficiently entrapped by increasing the initial GB concentration [22]. In all cases, significant differences between GB concentrations for the same AA concentration were found.

By increasing the pH to 9.0, the AA release performance was maximal for GA100 NPs (Figure 2b), not influenced by the imposed compositions. For GA175 and GA300 NPs, the AA release increased in comparison with that observed at pH 7.4 as a result of the GA charge inversion. RE values demonstrated the most effective release from those NPs prepared with the highest initial AA concentration (5 mg/mL) with RE values ranging between 20 and 60%. Differences became significant between GB concentrations at this AA concentration for both NPs. 

### 3.3. Biological Characterization

Biocompatibility is the ability of a material to act with an adequate host response in a specific situation [30]. The ambiguity of this term reflects the ongoing development of how biomaterials interact with the human body and how these interactions determine the clinical success of a medical device [31]. In this study, the degree of hemolysis and the erythrocyte binding were used to evaluate the possible toxicity at the hematological level. In vitro cytotoxicity assays evaluated the potential toxicity on representative skin cell lines in the presence of GB (AA)-GA NPs. In addition, the antioxidant properties of these NPs were evaluated in both cell types using two different chemical methods. GB(AA)-GA NPs prepared at the highest GB concentration (20 mg/mL) were selected to assess the biocompatibility of the proposed systems.

#### 3.3.1. Interaction with Erythrocytes

1. Hemocompatibility studies with GB (AA)-GA NPs

Following ISO 10993-4 concerning the biological evaluation of medical devices and their interactions with blood, an in vitro hemocompatibility assay was performed [32]. Under the assayed conditions, the determination of the degree of hemolysis produced by NPs by incubation with erythrocyte suspension was determined. Supplementary Material Figure S3a shows representative results as a function of the imposed composition. The effect of NPs on the hemolytic response was evaluated as the ratio 1:10 (*v*/*v*) between erythrocytes and NP dispersion, considering previous results with other gelatin-based NPs [20]. Hemolysis fluctuates slightly with the AA and GB concentration, and GA gel strength, with values lower than 2%, for almost all NP systems. Only in the case of discrete conditions, values can achieve 2–3%. Considering the standards for which NPs are classified as non-hemolytic (<2%), slightly hemolytic (with values 2–5%) and hemolytic (values > 5%) [33], it could be concluded that the proposed NPs showed a non-hemolytic character.

Changes in surface charge due to the presence of NPs or their components can cause erythrocytes to aggregate and increase their binding with other types of cells such as monocytes [34]. In this work, the effect of NPs on erythrocyte aggregation was evaluated. Supplementary Material Figure S3b shows representative results of optical microscopy evaluating the effect of the NP systems prepared at the highest concentration of both GB and AA, on the morphology and distribution of erythrocytes. No evident binding of erythrocytes was observed when incubating with NPs, independently of the GA gel strength.

2. Determination of the reversible capacity of GB (AA)-GA NPs against the peroxidation induced by AAPH

**Figure 3:**
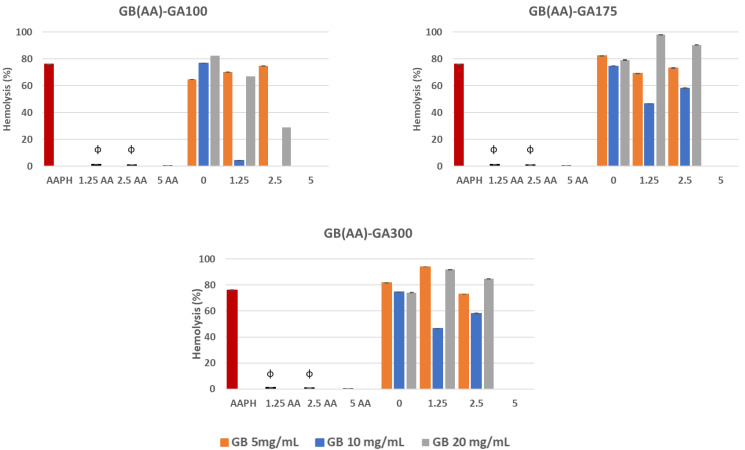
Reverse capability of GB(AA)-GA NPs against AAPH-induced hemolysis as a function of GA gel strength value and GB concentration. Results are expressed as the average of three independent experiments ± standard deviation. ^ϕ^ (*p* < 0.001) indicates significant differences between NPs and AA in solution for the same AA concentration.

Exposure of erythrocytes to free radicals could lead to changes in the membrane, including lipid peroxidation, reduced deformability, cell morphology, protein cross-linking, fragmentation, and hemolysis [35]. The generation of peroxyl radicals induced by the AAPH compound stands out. This causes the hemolysis of erythrocytes by the oxidation of proteins and lipids of the plasma membrane. APPH, can generate free radicals at a constant rate in PBS 7.4, by non-unimolecular thermal decomposition without the addition of potentially interfering cofactors and transition metals [36]. In this work, the interaction of AAPH with erythrocytes induced a percentage of hemolysis of 80%. The ability of GB(AA)-GA NPs and AA in solution at the equivalent concentration of those included in the NPs to reverse the adverse effects of peroxyl radicals generated by AAPH in erythrocytes was evaluated (Figure 3). Results demonstrated significant differences between AAPH-induced results and those in the presence of either AA in solution or gelatin-based NPs. 

The initial AA concentration was a crucial parameter of the antihemolytic effect of the gelatin-based NPs. Results demonstrated significant differences between bare NPs and AA-containing NPs. In all cases, NPs prepared at the highest AA concentration (5 mg/mL) showed the most prominent anti-hemolytic effect, for which the hemolysis could be completely prevented. When considering the effect of GB concentration, significant differences could be observed for all AA concentrations, except in the case of NPs containing the highest initial AA concentration (5 mg/mL), for which the maximal antihemolytic activity was detected. 

At this concentration, unlike the other values, there were no significant differences concerning those results obtained with AA in solution. Although AA in solution demonstrated total reversion of the AAPH-induced hemolysis during the assay, its low stability could assume a severe limitation. AA is an easily oxidized compound, especially under aerobic conditions and exposure to light, which is degraded in the first step, reversible to dehydroascorbic acid and then to oxalic acid in an irreversible way [27].

#### 3.3.2. Interaction with Representative Skin Cell Lines

1. Cell viability studies with GB (AA)-GA NPs

**Figure 4:**
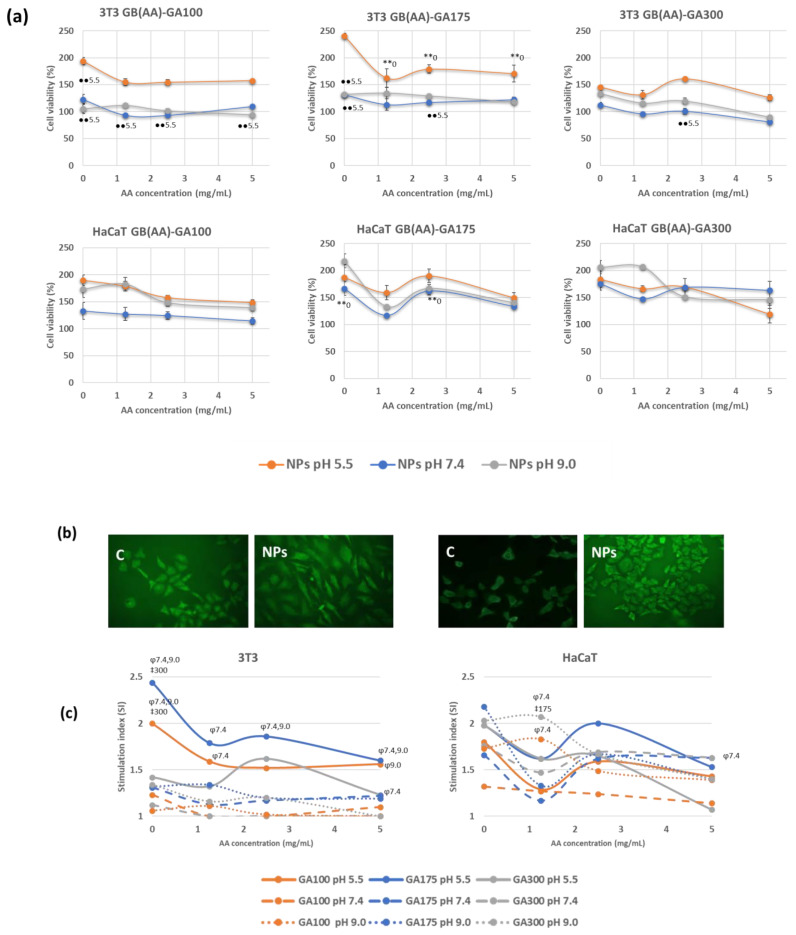
Effect of pH and the imposed compositions on cell viability response determined by MTT method (**a**), representative fluorescence microscopy images of cells incubated with bare NPs at pH 5.5 using the double staining AO/EtBr (**b**) and stimulation index values (**c**) on 3T3 and HaCaT cell lines incubated with GB(AA)-GA NPs, during 24 h. Results are expressed as the average of three independent experiments ± standard deviation. ** (*p* < 0.001) indicates significant differences between AA concentration for the same pH. ^●●^ (*p* < 0.001) indicates significant differences between pH for the same AA concentration. ^‡^ (*p* < 0.001) indicates significant differences between GA concentration for the same pH at a fixed AA concentration. ^φ^ (*p* < 0.001) indicates significant differences between pH for the same GA concentration at a fixed AA concentration.

Cytotoxicity assays are widely used to predict the potential toxicity of a substance in cell cultures [37]. Most of these tests are based on the viability of the cells and the direct relationship with the ability to perform physiological functions. In the present work, the evaluation of the acute toxicity of the NPs was assayed by the MTT method, which evaluates the cellular metabolic activity in living cells because it is dependent on NADPH [38]. Two representative cell lines from different skin layers were chosen. 3T3 cells are fibroblasts of murine origin found in the dermis, while HaCaT cells are human-derived keratinocytes from immortalized cells used in many differentiation studies. Both cell types are involved in the inflammation and proliferation phase of the normal healing process, and they showed modified functionality in chronic wounds. Figure 4a shows representative cell viability results in the presence of GB (AA)-GA NPs at pH 5.5. and those resulting in the increased pH to H 7.4 and 9.0 mimicking chronic wound environments. 

The response of 3T3 cells when incubated with NPs showed the highest cell viability at the corresponding pH of the healthy skin, with values that resulted to be a function of GA gel strength (Figure 4a). In the case of GA100 NPs, cell viability ranged between 150–200% were obtained at a pH of 5.5, in comparison with the other two pHs, at which cell viability values were close to 100%, Significant differences were found between viability values of GA100NPs at pH 5.5 and pH 7.4. Similar results were obtained in the case of GA175 NPs, with maximum cell viability at pH 5.5 (170–240%). Significant differences between results obtained between bare and AA-containing NPs for all the AA concentrations were found. By increasing pH, cell viability values ranged from 120–130% viability, slightly higher than those obtained with GA100 NPs. Significant differences with respect values at pH 5.5 were found in a few cases. In addition, GA300 NPs demonstrated lower differences between the incubating pHs, with cell viability values ranging from 90–160%. Significant differences were almost not found. HaCaT cells provided superior cell viability than 3T3 cells for the three considered pHs. In addition, significant differences were not found between results obtained at different incubation pHs. GA 100 NPs promoted very similar results at pH 5.5 and 9.0 (130–180%) and higher than those obtained a pH 7.4 (110–130%). The increase of GA gel strength (GA175 NPs or GA300 NPs) induced higher viability in an independent way of the pH (140–200%). 

Consequently, as a general trend, when incubated with the NPs systems, cell viability provided higher viability values than those observed with control cells, suggesting proliferation. This feature can be considered a promising characteristic since the proliferation of fibroblasts and keratinocytes during the proliferative and remodeling phases of wound healing leads to the first epidermal wound closure [39]. Cell viability values using the MTT assay, as a rough measure for cellular proliferation, together with the fluorescence microscopy (FM) studies using the double staining acridine orange/ethidium bromide (AO/EtBr) [40], demonstrated the proliferative effect induced by these gelatin-based NPs. Figure 4b shows representative FM of bare gelatin NPs at pH 5.5. 

To establish the effect of the imposed compositions on the induction of cell proliferation, the stimulation index (SI) was determined. Figure 4c shows representative results of the SI values on 3T3 and HaCaT cells. SI values showed signs to be a function of both cell type and pH. 3T3 fibroblasts showed maximum SI values at pH in the healthy skin, with values up to 2.5, decreasing to 1–1.5 at the representative pH of chronic wounds. Bare GA100 and GA175 NPs demonstrated significant differences with bare GA300 NPs at pH 5.5 at those corresponding to chronic wound conditions. In the presence of AA, only GA175 NPs demonstrated significant differences in SI values with pH. Keratinocytes showed to be less sensitive to pH changes, with SI values ranging between 1.5 and 2.2. Interestingly, NPs at pH 9.0, especially those prepared with the highest GA gel strength (GA300), were very effective in the induction of cell proliferation. Significant differences between pH 7.4 and 9.0 were found in discrete cases.

2. Protective capability of GB (AA)-GA NPs against oxidative stress induced by hydrogen peroxide

Further insights into the antioxidant capability of these NPs were determined by incubating cells with AA-containing NPs and inducing oxidative stress through the addition of hydrogen peroxide. The determination of cell viability in these conditions would provide information about the protective effect of these NPs against oxidative stress. Previously to these experiments, the optimal conditions for obtaining significant cell damage induced by H_2_O_2_ were determined. The ability to induce oxidative stress in the cells was assayed at two different H_2_O_2_ concentrations (1 and 4 mM). Results demonstrated that it is dependent on their concentration and the cell line. For an H_2_O_2_ concentration of 1 mM, the viability cellular decreased up to 79.9% in 3T3 and up to 87.8% in HaCaT. By increasing the concentration of H_2_O_2_ at 4 mM, viabilities decreased up to 33.4% in 3T3 and 52.6% in HaCaT.

**Figure 5:**
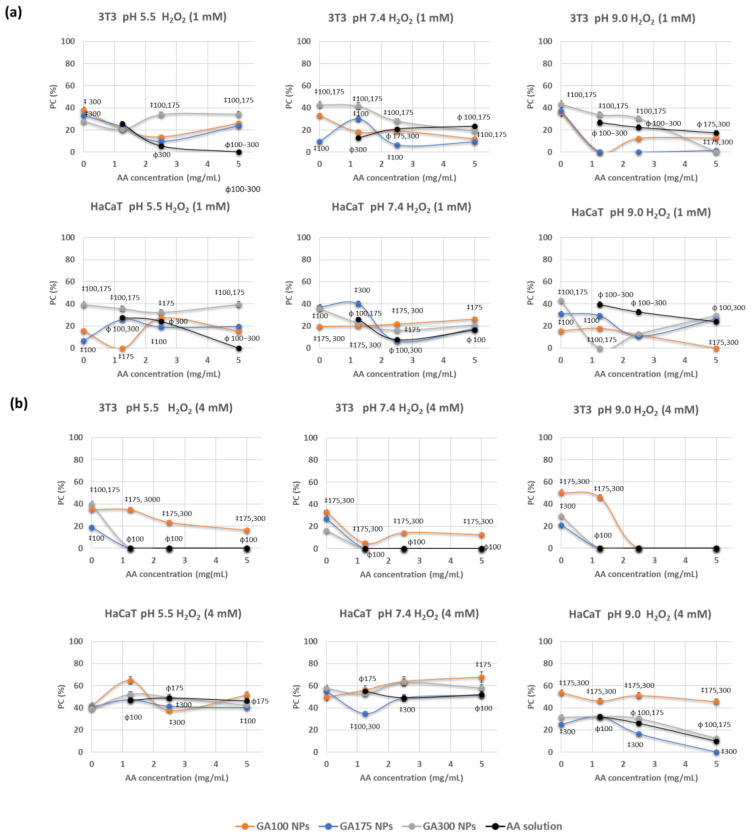
Protective capability (PC) of GB(AA)-GA NPs against oxidative stress induced by hydrogen peroxide at 1 mM (**a**) and 4 mM (**b**) on 3T3 and HaCaT cell lines on 3T3 and HaCaT cell lines during 24 h and determined by MTT method. Results are expressed as the average of three independent experiments ± standard deviation. ^‡^ (*p* < 0.001) indicates significant differences between GA concentration for the same pH at a fixed AA concentration. ^ϕ^ (*p* < 0.001) indicates significant differences between NPs and AA in solution for the same AA concentration.

Representative results of the protective capacity of the AA-containing NPs at the two oxidative strengths are shown in Figure 5. When considering the oxidative stress induced by 1 mM H_2_O_2_, results demonstrated to be dependent on the cell line, GA gel strength, and pH (Figure 5a). Hence, in the fibroblast cell line (3T3), significant differences were detected in the protective capabilities of GA300 NPs in comparison with other NPs, for almost all AA concentrations and pHs. Protective capacity (PC) values achieved 40%, with significant differences in comparison with AA in solution. Moreover, experiments carried out in the keratinocyte cell line (HaCaT) provided PE values more dependent on the imposed compositions. Hence, GA300NPs demonstrated to be very efficient at pH 5.5 with PE values close to 40% The increase of pH had a negative effect on the protective capabilities of the NPs being comparable or even lower than AA in solution, at pH 7.4 and 9.0, respectively. 

By inducing robust oxidative stress (4 mM H_2_O_2_), the protective course was strongly dependent on the studied variables (Figure 5b). When comparing the effect on the 3T3 cell line, AA in solution at any concentration and pH could reverse the induced oxidative stress. GA100 NPs provided the most efficient PC values, with maximum values of 30%. Significant differences were detected in comparison with GA175NPs and GA300NPs and AA in solution at all pH values. When considering the HaCaT cell line, GA100NPs proved their effectiveness again, at all pHs, but especially at pH 9.0, for which significant differences against AA in solution could be detected. PC values ranged from 40–60%.

## 4. Discussion

The procedure by which a drug is administered to treat a physiopathological alteration significantly affects its effectiveness. Administration of ascorbic acid to the skin through topical application remains a challenge due to its instability and aqueous solubility. To minimize the degradation of the drug and its loss of efficiency, encapsulation methods have been developed. The interaction of oppositely charged compounds between GB and GA enables the formation of GB(AA)-GA NPs with suitable physicochemical properties in terms of size, polydispersity, and efficiency of AA encapsulation (Figure 1). Although NPs are widely referenced in literature in treating chronic wounds, no evidence has been found regarding the optimal size range [41]. The skin presents several routes and limitations for its applicability as transdermal release systems; however, it becomes more permissive as it is damaged by UV radiation, or the presence of a pathogen, irritation, or a non-healing wound [42]. Moreover, LE values demonstrated that AA could be efficiently encapsulated in the proposed NPs with a degree of encapsulation comparable to previous synthetized gelatin-based NPs evolving other therapeutical molecules [20,21,22,23]. The evaluation of the in vitro release studies confirmed the working hypothesis. The pI of GA can be considered a controlling parameter for the formation of these NPs and the release of the encapsulated compound (Figure 2). The pI of GA, with values between 7 (low gel strength) and 9 (high gel strength), allows the formation of NPs at the pH of healthy skin and subsequent destabilization of them at pH values close to the chronic wound environment. 

During the development of any DDS, the biocompatibility of the systems or their starting products constitutes a key objective. Following EU rules, an in vitro hemocompatibility assay was performed. The obtained results allow us to conclude that NPs are hemocompatible, not producing either hemolysis or agglutination in human erythrocytes. These blood components were also used as a model to assay the APPH-induced oxidative stress (Figure 3). The antioxidant efficacy of NPs depends on the AA concentration, providing total reversion of the deleterious effect of AAPH at the highest AA concentration, and showing similar behavior to AA in solution. 

The biocompatibility assessment also included the determination of the cytotoxicity of the NPs and their starting products. Concerning the starting compounds, it should be noted that gelatin, given its extensive use in the fields of pharmacy, cosmetics, and food, is considered GRAS (Generally Regarded As Safe) by the FDA (Food Drug Administration). Gelatin has a low antigenicity compared to collagen and other proteins of animal origin due to the denaturation process it undergoes. In addition, gelatin does not generate hazardous by-products when subjected to enzymatic degradation [17]. Previous work in our lab demonstrated how gelatin did not induce in vitro cytotoxicity at concentrations up to 2 mg/mL [20]. Regarding AA, studies in the literature reported how high concentrations (20 mmol/L) of this compound can induce cell death of some cell lines, as it releases hydrogen peroxide, which can alter metabolism and redox balance [43], so it can cause decreased viability, and even cell death. Although these conditions could differ from those used in the present work, it should be noted that NPs, due to their special physicochemical properties that differentiate them from their starting products, could cause changes in cell morphology, metabolism, membrane integrity, and proliferation, at a smaller concentration than those corresponding to the starting compounds [44]. Consequently, the cytotoxicity of the whole DDS could be characterized. 

In this work, the toxicity of various NP systems was evaluated by representative skin cells such as fibroblasts and keratinocytes due to their direct involvement in the healing process. The cytotoxicity assay established the biocompatible nature of these NPs. Therefore, either in healthy skin conditions (pH 5.5), which takes place in the formation of these NPs, or in the representative pH of chronic wounds (pH 7.4 and 9.0), which are expected to induce the AA release, according to our working hypothesis (Figure 4) no cytoxicity was detected. It is well-known that close control of pH conditions is crucial for optimal culture conditions, and pH values in the range 7.2–7.4 are preferred for most cells. However, fibroblast-like cells seem to prefer slightly basic conditions (pH 7.4–7.7), while continuous transformed cell lines have a preference for more acid pH conditions (7.0–7.4) [45]. Proper control of the pH was performed by cell culture media containing phenol red and carbonate-based buffer. In this work, viability values seem to be maximal at pH 5.5, especially in the case of 3T3 cells, decreasing slightly by increasing the pH, but with significant differences only in a few cases and with viability values greater than 100% in all cases. The induced proliferation was evaluated in terms of SI values. Although results are sensitive to cell line and pH, this feature can be considered a promising characteristic since the proliferation of fibroblasts and keratinocytes during the proliferative and remodeling phases of wound healing leads to the first epidermal wound closure [39]. Importantly, gelatin introduces RGD (Arg-Gly-Asp tripeptide) sequences that have been reported to improve cell adhesion, migration, and proliferation of cells. The proliferative effect is mainly associated with the presence of gelatin as a component of these NPs. This protein, derived from the denaturation of collagen, binds to receptors and promotes the adhesion and proliferation of skin cells [46]. 

Nowadays, topical application of AA is often used as part of a wound care regimen that includes other interventions such as proper wound cleaning, debridement, infection control, and nutritional support. The dose of ascorbic acid in topical formulations can vary depending on the specific product and the severity of the wound. In some cases, topical ascorbic acid may be applied once or twice daily. In this work, AA incorporated in the gelatin-based NPs may provide an opportunity to increase the therapeutic effect of AA under chronic wound conditions. To patient compliance, AA would be protected and selectively released due to the charge reversal approach at the pH values of the chronic wound. Results on cell viability, with values overall 100% in all cases, ensure the lack of cytotoxicity under these conditions. Senescence fibroblasts and keratinocytes on chronic wounds would benefit from the stimulation of ECM production mediated by the gelatin-based carrier.

An excess of ROS characterizes the impaired healing wounds. Hydrogen peroxide was used in this work as a precursor of oxidative stress in the model cell lines. The protective capacity of these NPs resulted a function of the imposed compositions and the strength of induced cell damage (Figure 5). By low-inducing effect (1 mM H_2_O_2_), PE values were comparable to the values obtained with AA in solution, with GA300 NPs providing maximum PE values. By increasing the oxidative stress (4 mM H_2_O_2_), AA in solution showed minimal PE values, but GA100NPs efficiently modulated the protective capacity. The morphological and physiological properties of the cells could explain differences in their sensitivity and protective behavior. Accordingly, while keratinocytes are an example of cells representative of the epidermis, fibroblasts are found in the dermal skin layer [47]. For this reason, 3T3 cells are more sensitive than HaCaT cells to the deleterious effect of hydrogen peroxide, providing a more limited protective capability in the presence of NPs.

Wound healing requires a balance between the accumulation of collagen and non-collagen in the extracellular matrix and the remodeling of the matrix using matrix metalloproteinases (MMPs) and tissue MPP inhibitors (TIMPs). The combination of increased MMPs and decreased TIMPs in chronic wounds suggests that the proteolytic environment makes healing difficult. Both events avoid the scaffold formation needed for cell migration and prevent the ECM and granulation tissue formation. At the elevated levels present in chronic wounds, MMPs degrade not only nonviable collagen but also viable collagen. Therefore, collagen-based wound materials can be considered suitable to address elevated levels of MMPs by acting as a sacrificial substrate in the wound [48,49]. Ongoing research focuses on the role of these gelatin-based NPs on collagen growth and protease activity in mimicking chronic wound environments. 

## 5. Conclusions

The interaction of oppositely charged compounds between gelatin type A (100, 175, and 300 gel bloom) and gelatin type B (75 gel bloom) at the pH of healthy skin has been used to prepare AA-including NPs by associate phase separation. As a result, NPs with optimal values concerning size (100–200 nm), pdI (0.4) and LE (60%) values have been obtained. The increase of pH, mimicking the conditions of chronic wounds, has allowed the AA release from the NPs systems, especially with GA100NPs, taking into account the dependence of pI with the gel strength values of this protein. 

Hemocompatibility studies demonstrated that NPs are not hemolytic and do not induce agglutination under in vitro conditions. In addition, the capability of NPs to reverse the peroxidation induced by AAPH demonstrated total antihemolysis as a function of AA concentration. Representative cell lines from both epidermis (HaCaT cell line), and dermis (3T3 cell line) layers have been used to assess the in vitro cell viability induced by the NPs systems. Cytotoxicity has not been detected in any case and the induced proliferation was evaluated as an SI value (1.5–2.5 as a function of cell line and pH).

The antioxidant capacity of AA-containing NPs depends on both the degree of induced oxidation and the cell line. By inducing little oxidative stress (1 mM H_2_O_2_), both NPs and AA in solution can reverse the cell damage. However, by inducing more robust oxidative damage (4 mM H_2_O_2_), NPs but not AA in solution demonstrated their protective capacity, especially at the pH of chronic wounds. 

It can be deduced that GA at the lower gel strength (GA100) promotes the best results for the proposed applications. GA100 shows that having a pI close to 7 responds efficiently to the charge reversion at pH representative of chronic wounds under the work hypothesis. Although the effect of AA concentration became unclear in some cases, formulations including the highest AA concentration (5 mg/mL) would be considered optimal to achieve optimal therapeutical responses. 

In conclusion, the proposed gelatin-based NPs showed promising properties as biocompatible inductors of proliferation in both fibroblast and keratinocyte cell lines. Differences in ionization properties of GA as a function of gel strength make them controlling parameters in the in vitro AA release and reversible/protective antioxidant characteristics of AA-containing NPs in erythrocytes and representative skin cell lines under conditions that mimic chronic wounds.

## Data Availability

Data are contained within the article and Supplementary Materials.

## Supplementary Materials

The following supporting information can be downloaded at: https://www.mdpi.com/article/10.3390/antiox13030299/s1, Figure S1: Concentration-dependence DCPIP-AA calibration curves at pH 5.5, 7.4 and 9.0, Figure S2. Effect of the imposed compositions on the size (a), pdI (b) and loading efficiency of AA (c) of GB(AA)-GA NPs, Figure S3: Relative hemolysis (a) and representative optical microscopy of erythrocytes (b) after incubation of gelatin-based NPs.
