# Supplementary material for: Insight into the Antioxidant Activity of Ascorbic Acid-Containing Gelatin Nanoparticles in Simulated Chronic Wound Conditions
Source: Antioxidants (Basel). 2024 Feb 28;13(3):299. doi: 10.3390/antiox13030299 (PMC10967451; doi:10.3390/antiox13030299)
Supplement: Supplementary file 1 [file antioxidants-13-00299-s001.zip › antioxidants-2846320-supplementary.pdf]

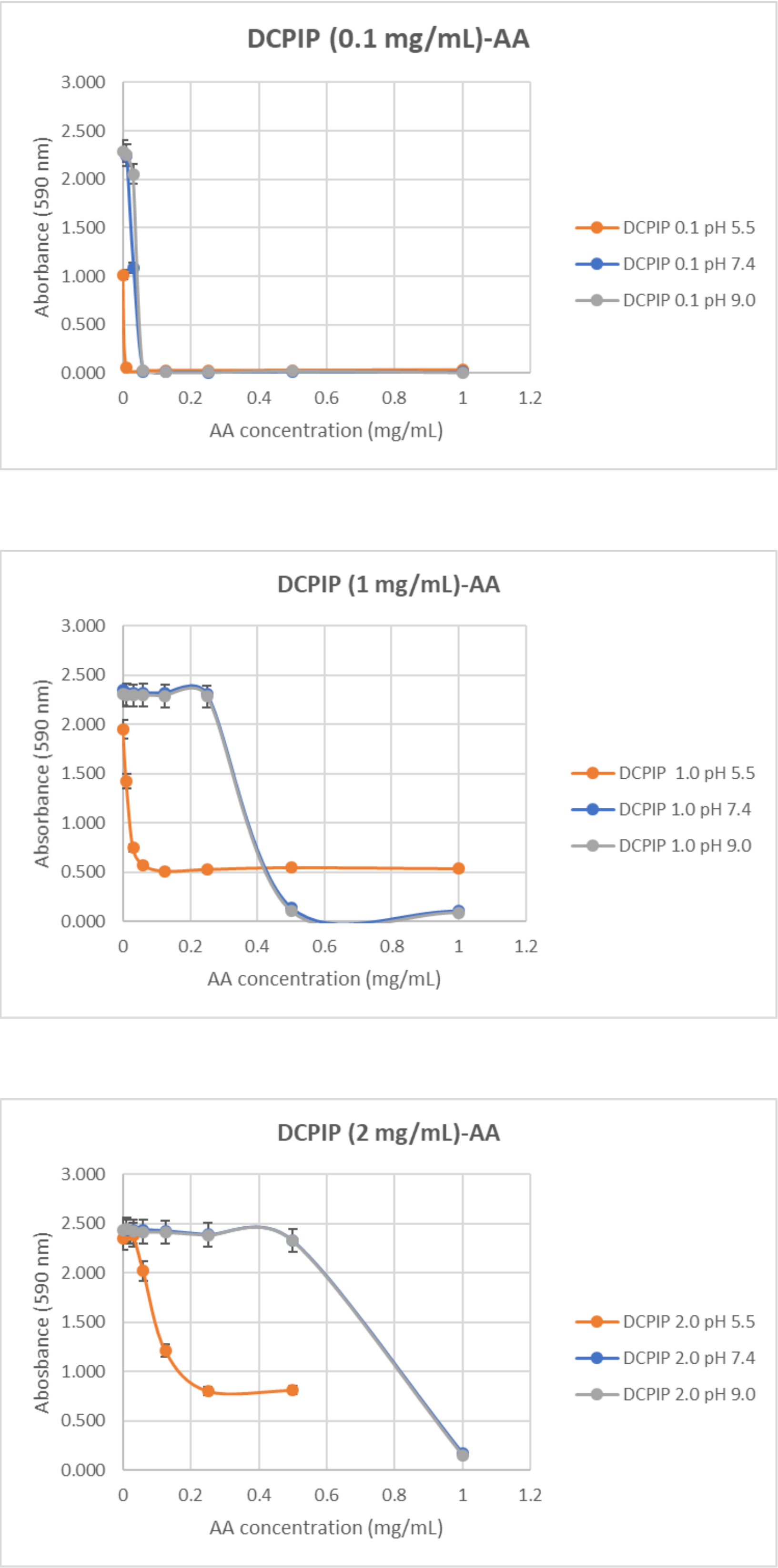

Figure S1. Concentration dependence DCPIP-AA calibration curves at pH 5.5, 7.4 and 9.0. Results are expressed as the average of three independent experiments ± standard deviation.

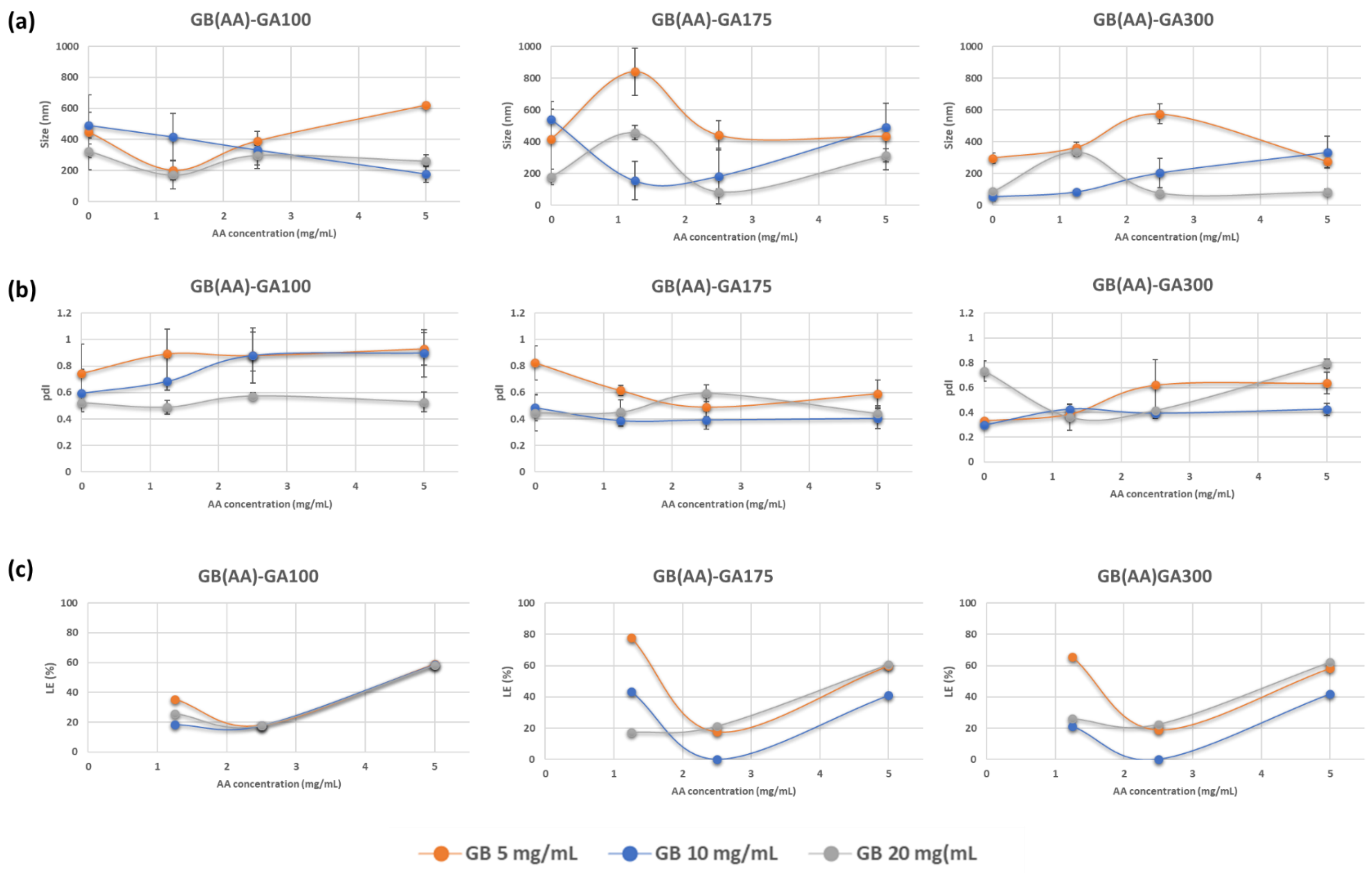

Figure S2. Effect of the imposed compositions on the size (**a**), pdI (**b**) and loading efficiency of AA (**c**) of GB(AA)-GA NPs. Results are expressed as the average of three independent experiments ± standard deviation.

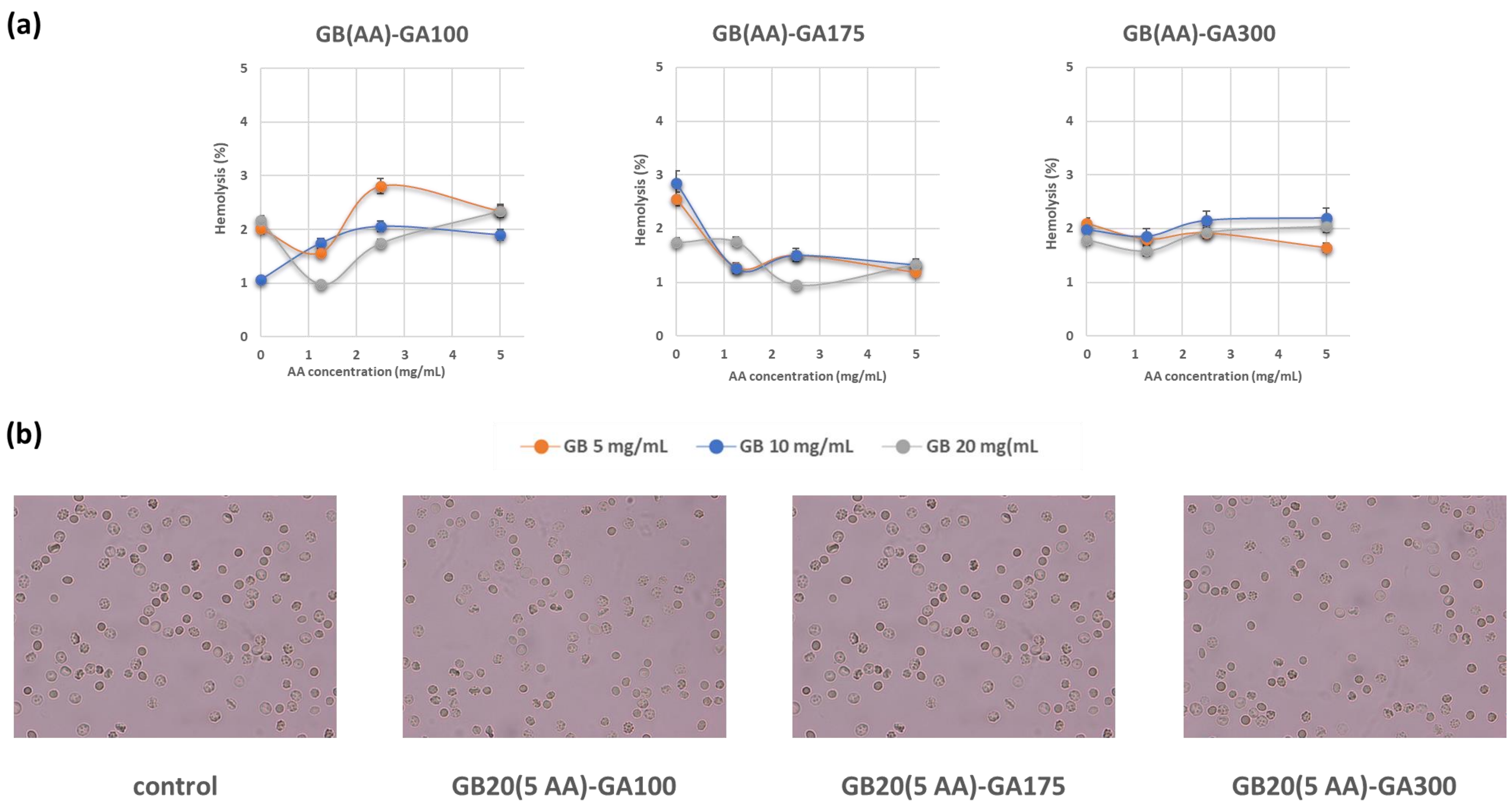

Figure S3. Relative hemolysis (**a**) and representative optical microscopy of erythrocytes (**b**) after incubation of gelatin-based NPs prepared at the highest concentration of both GB (20 mg/mL) and AA (5 mg/mL). Results are expressed as the average of three independent experiments ± standard deviation.
